# Alpha-tocotrienol is the most abundant tocotrienol isomer circulated in plasma and lipoproteins after postprandial tocotrienol-rich vitamin E supplementation

**DOI:** 10.1186/1475-2891-11-5

**Published:** 2012-01-17

**Authors:** Syed Fairus, Rosnah M Nor, Hwee M Cheng, Kalyana Sundram

**Affiliations:** 1 Malaysian Palm Oil Board (MPOB), 6, Persiaran Institusi, Bandar Baru Bangi, 43000, Kajang, Selangor, Malaysia; 2 Faculty of Medicine, University Malaya, 50603, Kuala Lumpur, Malaysia; 3 Malaysian Palm Oil Council (MPOC), 2nd Floor, Wisma Sawit, Lot 6, SS6, Jalan Perbandaran, 47301, Kelana Jaya, Selangor, Malaysia

**Keywords:** Tocotrienols, Tocopherols, Vitamin E, Postprandial plasma lipoproteins

## Abstract

**Background:**

Tocotrienols (T3) and tocopherols (T), both members of the natural vitamin E family have unique biological functions in humans. T3 are detected in circulating human plasma and lipoproteins, although at concentrations significantly lower than α-tocopherol (α-T). T3, especially α-T3 is known to be neuropotective at nanomolar concentrations and this study evaluated the postprandial fate of T3 and α-T in plasma and lipoproteins.

**Methods:**

Ten healthy volunteers (5 males and 5 females) were administered a single dose of vitamin E [526 mg palm tocotrienol-rich fraction (TRF) or 537 mg α-T] after 7-d pre-conditioning on a T3-free diet. Blood was sampled at baseline (fasted) and 2, 4, 5, 6, 8, and 24 h after supplementation. Concentrations of T and T3 isomers in plasma, triacylglycerol-rich particles (TRP), LDL, and HDL were measured at each postprandial interval.

**Results:**

After TRF supplementation, plasma α-T3 and γ-T3 peaked at 5 h (α-T3: 4.74 ± 1.69 μM; γ-T3: 2.73 ± 1.27 μM). δ-T3 peaked earlier at 4 h (0.53 ± 0.25 μM). In contrast, α-T peaked at 6 h (30.13 ± 2.91 μM) and 8 h (37.80 ± 3.59 μM) following supplementation with TRF and α-T, respectively. α-T was the major vitamin E isomer detected in plasma, TRP, LDL, and HDL even after supplementation with TRF (composed of 70% T3). No T3 were detected during fasted states. T3 are detected postprandially only after TRF supplementation and concentrations were significantly lower than α-T.

**Conclusions:**

Bio-discrimination between vitamin E isomers in humans reduces the rate of T3 absorption and affects their incorporation into lipoproteins. Although low absorption of T3 into circulation may impact some of their physiological functions in humans, T3 have biological functions well below concentration noted in this study.

## Background

Vitamin E is the generic name for a group of 8 plant-derived, lipid soluble substances ("tocols") including four tocopherol (T) and four tocotrienol (T3) derivatives. T3 are similar to T in molecular structure, except that they have an isoprenoid tail with three unsaturation points instead of a saturated phytyl chain. Vitamin E is a recognized antioxidant and thought beneficial for human health. There have been several indications that T3 may result in superior therapeutic properties compared to T [1-8].

The absorption and biokinetics of T3 in humans are however not fully understood. *Inter alia*, the above issues related to the absorption and biokinetics have been linked to several findings relating the physiological outcomes of T3 [9-12]. In comparison to α-T, the metabolic pathways relevant to T3 have hardly been elucidated and optimized. Several human studies have investigated the absorption of T3 into circulating plasma [9-11,13-21] and lipoproteins [2,19,20]. The detection of T3 in plasma and lipoprotein fractions has proven difficult, possibly due to its low occurrence. In comparison to α-T, concentrations of T3 were significantly lower. Rapid disappearance of these T3 has raised questions about their potential as potent lipid-soluble antioxidant. This is most probably one of the reasons why T3 are given a low score for their biological vitamin E activity compared to α-T [22].

T as well as T3 are transported within lipoprotein particles while circulating in the blood, but their distribution in lipoproteins has been documented only occasionally. Most studies investigating absorption of T3 in the human circulatory system focused on the plasma content of T3. From these limited human studies [2,19,20], distribution of T3 in lipoproteins was significantly lower than that of α-T. Following postprandial intervention, T3 transport in lipoproteins appears to follow complex biochemically mediated pathways within the lipoprotein cascade [19]. The mechanism of T3 transport in human lipoproteins has not been conclusively investigated and discussed. Although laboratory evidence has been very promising [5,8], T3 supplementation in humans has produced inconsistent results [6,7]. In addition, most studies investigating the response of T3 supplements, investigated only total plasma concentration of T3 [9-13,16,17] and rarely in various lipoprotein fractions [19,20].

Response of plasma and lipoproteins to T3 may be determined by the dose of T3 supplementation. This additionally could influence the fasting T3 level in blood [14]. Since supplementation with high dose of T3 has demonstrated that T3 were detected in plasma, TRP, LDL, and HDL following 8-h of postprandial challenge [19], it is intriguing to investigate whether lower dose of T3 supplementation would also resulted a similar observation. Since α-T is the most bio-active form of vitamin E [22], it is crucial to refer to α-T when comparing the biological activity of other isomers of vitamin E including T3. With this in mind, we investigated the metabolic fate of T3 as well as α-T in plasma and lipoprotein fractions in normolipemic humans through the current postprandial study.

## Methods

### Subjects

10 volunteers (5 males and 5 females) who were employees of the Malaysian Palm Oil Board (MPOB) were recruited for the study. Each volunteer was briefed on the objectives, design and protocol of the study before signing a consent form. The study was approved by the institutional ethics committee. All volunteers were normolipemic, nonsmokers, and did not show any clinical symptoms associated with lipid-related cardiovascular disease. Through the administration of a questionnaire and dietary interview, we established that none of the volunteers consumed any vitamin or herbal supplements nor were they taking any prescribed medication. Female volunteers were not pregnant, lactating, or taking contraceptives at the time of enrollment. The study was completed with the following baseline characteristics of the 10 volunteers: (mean ± SD): age, 23.8 ± 5.53 y; body mass index, 20.4 ± 1.83 kg/m^2^; plasma total cholesterol (TC), 4.08 ± 0.92 mmol/L; and plasma total triacylglycerol (TAG), 1.05 ± 0.34 mmol/L.

### Study design

The study was designed to elucidate the absorption and metabolic fate of palm T3 administered to humans in a postprandial model system, and compared to that of α-T at a similar dose. The dose selected was approximately 500 mg of vitamin E or 50% of the Tolerable Upper Limit Intake (UL) in humans [22]. The study was conducted in accordance to procedures published previously [19]. Volunteers were conditioned on a standardized fat-controlled diet (comprising breakfast, lunch and afternoon high tea) during a run-in period lasting 7 days for each rotation of the postprandial trial. Using a cross-over design, volunteers were subjected to two rotations whereby α-T and palm T3 rich-fraction (TRF) supplements were administered separately. One week wash-out period was allowed between each rotation. Meals were cooked with corn oil as the dietary fat source, and the same menu was repeated for each rotation. Daily food samples were duplicated and analysed for composition of fat and vitamin E. Content of fat and total vitamin E in the standardized fat- controlled diet was 48.0 ± 12.2 g/d and 14.9 ± 8.2 mg/d (4.7 ± 2.7 mg α-T/d, 9.6 ± 5.2 mg γ-T/d, and 0.6 ± 0.3 mg δ-T/d), respectively.

### Postprandial event

Volunteers fasted overnight (≥ 10 h) and reported to the laboratory on the next morning. After their body weight was recorded, 12 mL blood was drawn for a fasting, baseline sample (0 h). The volunteers then consumed the standardized test breakfast cooked with corn oil, which included a weighed portion of fried rice, fried potatoes, a slice of papaya, and tea. The test breakfast contained 30.5 ± 8.2 g fat and 10.7 ± 1.7 mg total vitamin E (3.2 ± 0.5 mg α-T, 7.1 ± 1.2 mg γ-T, and 0.5 ± 0.07 mg δ-T). The volunteers were then challenged with the vitamin E preparations: palm T3-rich fraction (TRF) or α-T. For the TRF treatment, 4 capsules of TRF (obtained in-house from the Agro Product Unit, MPOB) were used to provide a total of 526 mg vitamin E (α-T, 167 mg; α-T3, 157 mg; β-T3, 15.2 mg; γ-T3, 141.8 mg; δ-T3, 45.2 mg). For α-T treatment, 2 capsules of *RRR*-α-T (Natopherol^®^, Abbot Laboratories, Australia) were used to provide 537 mg vitamin E solely as α-T. This entire exercise was completed within 20 min of the first (baseline, 0 h) blood sampling. Blood samples were taken postprandially at 2, 4, 5, 6 and 8 h after the meal and vitamin E supplement were consumed. During this postprandial challenge, volunteers abstained from consuming any food and were only allowed to consume mineral water. They also refrained from any strenuous activity within these intervals. Following the end of the 8-h postprandial blood sampling, the volunteers were provided a full cooked meal with fat component contributed solely by corn oil. Late in the evening, they also consumed supper in their homes. On the next day after an overnight fast, a fasted blood sample was again drawn from each volunteer to complete the 24-h time point.

### Blood sampling and handling

Following blood collection into collection tubes containing EDTA (BD Vacutainer, Franklin Lakes, NJ), plasma was isolated by centrifugation at 3000 × g for 20 min at 4°C. A fresh, 3 mL of recovered plasma was refrigerated overnight at 4°C and subsequently used for preparation of lipoprotein fractions; triacylglycerol-rich particles (TRPs), LDL, and HDL. These lipoproteins were isolated from plasma by sequential ultracentrifugation using a 50.4 Ti rotor (Beckman Instruments Inc, Palo Alto, CA), as described previously [19,23]. The remaining plasma samples were aliquoted and snap-frozen in liquid nitrogen and stored at -80°C until analysed.

### Biochemical determinations

#### Plasma total cholesterol and triacylglycerol

Plasma lipids were analysed by enzymatic procedures using a Roche-Hitachi 902 Clinical Autoanalyzer (Roche-Hitachi, Japan) with reagents, calibrators, and controls supplied by Roche Diagnostics GmbH, IN.

#### Vitamin E analysis in plasma and lipoprotein fractions

Plasma and lipoprotein fractions (TRP, LDL, HDL) were extracted for vitamin E and analysed by HPLC as described previously [19,24]. The system used was an Agilent 1100 Series (Agilent Technologies Inc, Waldbrohn, Germany). Two normal-phase 5-μm silica columns (4.6 × 250 mm; Agilent Zorbax Rx-SIL, Agilent Technologies Inc, Palo Alto, CA) were fitted in series to enhance the separation of all vitamin E isomers, with a mobile phase consisting of hexane-isopropanol (flow rate of 2 mL/min, pressure of 133 bar, run time of 25 min). Identification of the vitamin E isomers was done using a fluorescence detector (Agilent 1100 Series, Agilent Technologies), with excitation at 295 nm and emission at 330 nm, as described previously [19].

### Statistical analysis

Postprandial responses were compared with the corresponding baseline value (0 h) and their trend was analysed by using repeated-measures analysis of variance (ANOVA). Changes were calculated as the difference between responses at each postprandial interval and baseline. Postprandial effects between treatments on plasma profiles were analysed for their time × treatment interaction by using two-factor repeated-measures ANOVA, whereas postprandial effects on lipoprotein profiles were analysed for their time × treatment × group (lipoproteins) interactions by using three-factor repeated-measures multiple analysis of variance (MANOVA). Area under the curve (AUC), which was defined as the total postprandial vitamin E response for the 24-h period, was also determined with the area normalized to the baseline concentration. Wilcoxon's signed-ranks test was performed to detect any significant difference between variables of interest. Results were presented as the mean ± SEM. Statistical analyses were performed by using SPSS for WINDOWS (version 11.0; SPSS Inc, Chigaco, IL), and significance was set at *P *< 0.05.

## Results

### Postprandial lipid responses

Following both α-T and TRF treatments, no significant changes were observed in plasma total cholesterol and triacylglycerol concentrations (Table 1). No significant changes in all lipid responses were observed between treatments.

**Table 1:** Plasma total cholesterol and triacylglycerol concentrations (mmol/L) following α-tocopherol and tocotrienol-rich fraction (TRF) treatments (Mean values ± SEM, n = 10).

	Total cholesterol (mmol/L)		Triacylglycerol (mmol/L)	
	**α-T**	**TRF**	**α-T**	**TRF**

0 h*	4.29 ± 0.19	4.45 ± 0.23	0.96 ± 0.04	1.05 ± 0.08
2 h	4.28 ± 0.23	4.34 ± 0.35	1.22 ± 0.09	1.30 ± 0.13
4 h	4.15 ± 0.24	4.33 ± 0.27	1.41 ± 0.15	1.37 ± 0.14
5 h	4.24 ± 0.26	4.33 ± 0.29	1.37 ± 0.14	1.29 ± 0.13
6 h	4.39 ± 0.23	4.54 ± 0.37	1.23 ± 0.07	1.31 ± 0.16
8 h	4.37 ± 0.25	4.58 ± 0.37	0.95 ± 0.06	1.02 ± 0.13
24 h	4.49 ± 0.23	4.35 ± 0.28	0.90 ± 0.05	0.91 ± 0.08
AUC 24 h	105.11 ± 5.53	106.78 ± 6.78	24.47 ± 1.37	25.35 ± 2.25

α-T, alpha-tocopherol; TRF, tocotrienol-rich fraction; AUC, area under the curve (arbitrary units).

* No significant differences between the treatments at baseline (0 h) were found for either total cholesterol or triacylglycerol (Wilcoxon's signed-ranks test).

### Postprandial plasma tocopherols responses

α-T was the predominant vitamin E isomer detected in plasma throughout the entire postprandial intervals following both treatments. Plasma α-T concentrations increased significantly starting from 4 h, before peaking at 8 h (37.8 ± 3.59 μM) and 6 h (30.13 ± 2.91 μM) after supplementation with α-T and TRF, respectively (Table 2). A similar pattern was reflected for plasma γ-T, which increased significantly from 4 h, peaked at 5 h (1.64 ± 0.15 μM) after α-T treatment, and 6 h (2.15 ± 0.41 μM) after TRF treatment. There was no significant time × treatment interaction of α-T and γ-T concentrations between both treatments. However, plasma total α-T concentrations throughout the postprandial period (24 h) were significantly higher after the α-T treatment compared to that of TRF treatment (measured as the AUC).

**Table 2:** Plasma tocopherols (T) concentrations after supplementation with the α-tocopherol or tocotrienol-rich fraction (TRF) treatments (Mean values ± SEM, n = 10).

	α-T			TRF	
			
	α-T	γ-T		α-T	γ-T
			μM		
0 h*	23.38 ± 1.03	0.36 ± 0.12		22.95 ± 1.26	0.35 ± 0.15
2 h	26.31 ± 1.81	0.63 ± 0.15		22.98 ± 1.20	0.57 ± 0.18
Change^†^	2.90 ± 1.63	0.29 ± 0.14		0.05 ± 0.65	0.22 ± 0.17
4 h	32.65 ± 2.49^‡^	1.42 ± 0.12^‡^		26.45 ± 1.89^‡^	1.70 ± 0.28^‡^
Change	9.26 ± 1.00	1.06 ± 0.17		3.51 ± 1.23	1.34 ± 0.34
5 h	36.38 ± 3.17^‡^	1.64 ± 0.15^‡^		28.15 ± 2.61^‡^	1.96 ± 0.40^‡^
Change	12.98 ± 2.79	1.27 ± 0.22		5.20 ± 1.93	1.61 ± 0.46
6 h	37.4 ± 3.51^‡^	1.57 ± 0.17^‡^		30.13 ± 2.91^‡^	2.15 ± 0.41^‡^
Change	14.00 ± 3.25	1.22 ± 0.19		7.17 ± 2.18	1.80 ± 0.48
8 h	37.8 ± 3.59^‡^	1.48 ± 0.27^‡^		29.79 ± 2.30^‡^	1.76 ± 0.22^‡^
Change	14.39 ± 3.32	1.13 ± 0.29		6.85 ± 1.39	1.42 ± 0.29
24 h	32.92 ± 3.58	0.35 ± 0.16		26.79 ± 1.81	0.20 ± 0.11
Change	9.52 ± 3.41	-0.02 ± 0.17		3.83 ± 1.07	-0.14 ± 0.19
AUC 24 h	353.52 ± 30.40^§^	9.92 ± 1.68		286.16 ± 19.85^§^	11.10 ± 1.47

T, tocopherols; TRF, tocotrienol-rich fraction; AUC, area under the curve (arbitrary unit); α-T, alpha-tocopherol; γ-T, γ-tocopherol.

* No significant differences in plasma α-T and γ-T concentrations at baseline (0 h) were found between the treatments.

† Changes were calculated as the difference between values at each postprandial interval and baseline (0 h).

‡ Significant increment from baseline (0 h) value, *P *< 0.05 (Wilcoxon's signed-ranks test).

§ Significant difference of AUC for α-T between the treatments, *P *< 0.05 (Wilcoxon's signed-ranks test).

### Postprandial plasma tocotrienols responses

**Figure 1:**
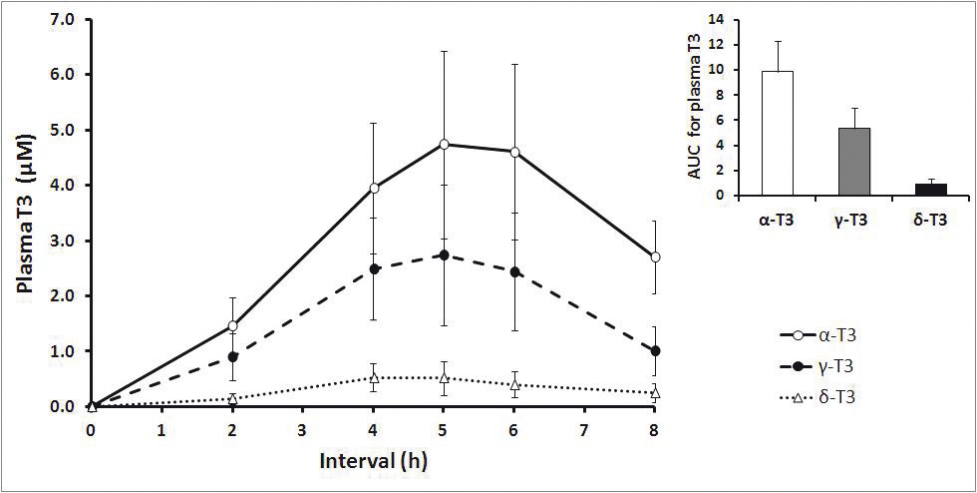
**Mean (± SEM) plasma tocotrienols (T3) concentrations in healthy volunteers (n = 10) after supplementation with the tocotrienol-rich fraction (TRF)**. α-T3, α-tocotrienols (○); γ-T3, γ-tocotrienols (●) and δ-T3, δ-tocotrienols (Δ) were detected in plasma starting from 2 h until 8 h. No T3 were detected at 0 h and 24 h. Inset: AUC, area under the curve, 0-8 h (arbitrary units) is presented as means ± SEM (n = 10) for α-T3, γ-T3 and δ-T3.

Unlike during α-T treatment, supplementation with TRF resulted in the incorporation of T3 into plasma and changes in the vitamin E composition. Following TRF treatment, α-T3, γ-T3 and δ-T3 were detected in postprandial plasma (Figure 1), along with α-T and γ-T. However, concentration of α-T3 (1.46 ± 0.52 to 4.74 ± 1.69 μM), γ-T3 (0.90 ± 0.42 to 2.73 ± 1.27 μM) and δ-T3 (0.14 ± 0.10 to 0.53 ± 0.25 μM) was significantly lower compared to that of α-T (22.95 ± 1.26 to 30.13 ± 2.91 μM), even when volunteers were supplemented with the tocotrienol-rich TRF. T3 were not detected in fasting plasma samples (0 h) or 24 h after supplementation with TRF. All T3 isomers increased postprandially starting from 2 h and thereafter peaked at 5 h (α-T3, 4.74 ± 1.69 μM; γ-T3, 2.73 ± 1.27 μM) before declining from 6 h onwards. δ-T3 was however peaked earlier at 4 h (0.53 ± 0.25 μM).

α-T3 was the major T3 isomer detected throughout the whole postprandial period.

### Distribution of tocopherols in lipoprotein fractions

The distribution of α-T and γ-T in TRP, LDL, and HDL was expressed as percentage of plasma total vitamin E (Table 3). Concentration of α-T in all lipoprotein fractions was significantly higher than γ-T during both treatments. However, only γ-T in LDL and HDL increased significantly from its baseline (0 h) values. Following TRF treatment, γ-T in LDL increased significantly from 4 h to 8 h, whereas after α-T treatment, LDL γ-T increased significantly only at 8 h. In HDL, only γ-T increased significantly from its baseline value (from 4 h to 8 h) after supplementation with TRF. No significant increment in postprandial α-T and γ-T in TRP were observed. In TRP, between 17.39 ± 1.72% to 33.34 ± 3.34%, and 17.96 ± 4.60% to 26.93 ± 3.53% of total circulating plasma vitamin E was detected as α-T during α-T and TRF treatments, respectively. α-T was significantly higher in TRP (from 2 h to 6 h) following a-T treatment versus the TRF treatment.

**Table 3:** Distribution of tocopherols (T) in plasma lipoprotein fractions during supplementation with the α-tocopherol and tocotrienol-rich fraction (TRF) treatments (mean values ± SEM, n = 10)*

	α-T						γ-T					
	
	TRP		LDL		HDL		TRP		LDL		HDL	
	
	α-T	TRF	α-T	TRF	α-T	TRF	α-T	TRF	α-T	TRF	α-T	TRF
	**%**						**%**					
0 h	18.21 ± 2.31	21.2 ± 4.79	39.53 ± 2.53	41.25 ± 3.67	40.73 ± 2.57	36.20 ± 2.84	0.08 ± 0.08	0.33 ± 0.21	0.46 ± 0.19	0.47 ± 0.21	1.00 ± 0.41	0.55 ± 0.24
2 h	29.85 ± 3.57	25.13 ± 4.02	33.48 ± 2.56	33.53 ± 3.80	34.34 ± 2.50	30.87 ± 2.87	0.79 ± 0.22	0.97 ± 0.41	0.51 ± 0.15	0.58 ± 0.23	1.02 ± 0.27	0.76 ± 0.23
Change^†^	11.65 ± 2.44^§^	3.93 ± 1.24^§^	-6.05 ± 1.66	-7.72 ± 2.58	-6.38 ± 1.71	-5.34 ± 1.10	0.72 ± 0.22	0.64 ± 0.31	0.05 ± 0.17	0.11 ± 0.27	0.01 ± 0.25	0.22 ± 0.29
4 h	33.34 ± 3.34	26.93 ± 3.53	30.74 ± 2.33	26.38 ± 2.78	31.78 ± 2.50	26.18 ± 2.89	2.02 ± 0.31	2.27 ± 0.54	0.80 ± 0.13	1.06 ± 0.18^‡^	1.32 ± 0.19	1.52 ± 0.22^‡^
Change	15.14 ± 2.83^§^	5.73 ± 1.66^§^	-8.79 ± 2.22	-14.48 ± 2.49	-8.95 ± 1.20	-10.02 ± 2.11	1.94 ± 0.34	1.95 ± 0.51	0.35 ± 0.21	0.59 ± 0.23	0.32 ± 0.34	0.97 ± 0.30
5 h	31.55 ± 3.29	26.40 ± 3.92	31.89 ± 2.13	25.97 ± 3.00	32.01 ± 2.34	26.12 ± 3.11	2.06 ± 0.39	2.37 ± 0.54	0.97 ± 0.08	1.19 ± 0.10^‡^	1.38 ± 0.20	1.61 ± 0.27^‡^
Change	13.34 ± 2.77^§^	5.20 ± 1.72^§^	-7.64 ± 1.58	-15.29 ± 3.12	-8.72 ± 1.75	-10.09 ± 2.01	1.99 ± 0.42	2.04 ± 0.49	0.51 ± 0.25	0.72 ± 0.20	0.38 ± 0.38	1.06 ± 0.35
6 h	27.76 ± 2.14	24.10 ± 4.29	36.27 ± 1.97	29.45 ± 3.47	31.58 ± 2.27	25.50 ± 2.52	1.81 ± 0.40	2.30 ± 0.59	1.04 ± 0.09	1.39 ± 0.16^‡^	1.40 ± 0.25	1.70 ± 0.22^‡^
Change	9.55 ± 2.07^§^	2.90 ± 1.78^§^	-3.26 ± 1.65	-11.80 ± 2.64	-9.15 ± 1.48	-10.71 ± 2.37	1.73 ± 0.43	1.97 ± 0.54	0.58 ± 0.28	0.92 ± 0.23	0.40 ± 0.37	1.15 ± 0.28
8 h	17.93 ± 2.24	17.96 ± 4.60	40.41 ± 1.83	35.74 ± 3.46	37.54 ± 1.99	31.26 ± 2.82	1.07 ± 0.35	1.36 ± 0.44	1.28 ± 0.20^‡^	1.62 ± 0.13^‡^	1.64 ± 0.39	1.97 ± 0.30^‡^
Change	-0.28 ± 2.15	-3.24 ± 1.85	0.88 ± 1.64	-5.52 ± 1.34	-3.18 ± 1.73	-4.95 ± 2.23	1.00 ± 0.37	1.04 ± 0.33	0.83 ± 0.34	1.14 ± 0.28	0.64 ± 0.36	1.43 ± 0.41
24 h	17.39 ± 1.72	19.94 ± 4.46	40.71 ± 2.38	38.82 ± 3.60	40.59 ± 2.11	40.42 ± 3.12	0.12 ± 0.09	0.21 ± 0.21	0.33 ± 0.19	0.18 ± 0.12	0.87 ± 0.45	0.43 ± 0.22
Change	-0.82 ± 2.35	-1.26 ± 2.11	1.17 ± 2.27	-2.43 ± 1.92	-0.15 ± 1.16	4.22 ± 1.35	0.05 ± 0.10	-0.12 ± 0.88	-0.13 ± 0.22	-0.18 ± 0.23	-0.13 ± 0.49	0.01 ± 0.34
AUC	501.58 ± 42.79	495.54 ± 101.10	928.26 ± 40.04	805.20 ± 73.76	899.04 ± 45.72	806.29 ± 62.67	20.07 ± 5.26	25.37 ± 7.97	19.38 ± 2.92	22.53 ± 2.10	30.25 ± 7.60	29.72 ± 5.02

TRF, tocotrienol-rich fraction; TRP, triacylglycerol rich particles; AUC, area under the curve (arbitrary unit); α-T, α-tocopherol; γ-T, γ-tocopherol.

* Significant time × treatment × group (lipoproteins) interaction of α-T were found (3-factor repeated-measures MANOVA).

† Changes were calculated as the difference between values at each postprandial interval and baseline (0 h).

‡ Significant increment from baseline (0 h) value, *P *< 0.05 (Wilcoxon's signed-ranks test).

§ Significant difference of α-T content in TRP between α-tocopherol and TRF treatments, *P *< 0.05 (Wilcoxon's signed-ranks test)

### Distribution of tocotrienols in lipoprotein fractions

**Figure 2:**
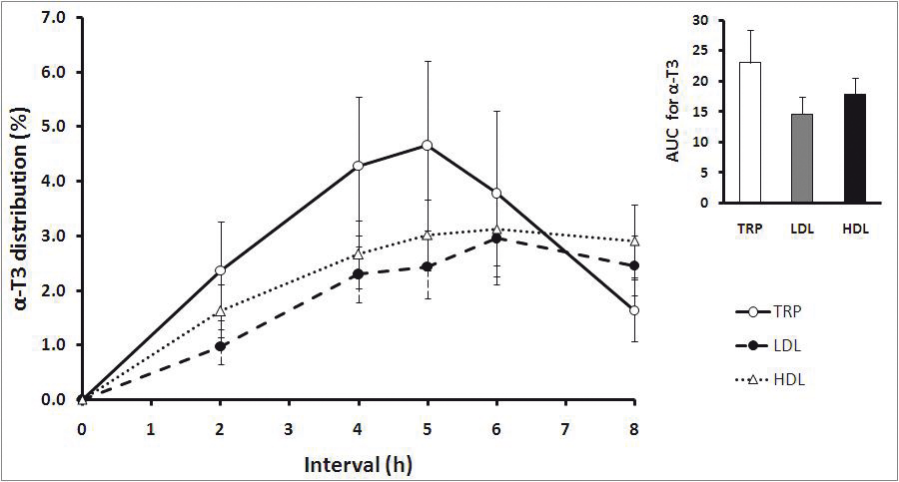
**Mean (± SEM) α-tocotrienols (α-T3) distributions in triacylglycerol rich particles, TRP (○); LDL (●); and HDL (Δ) of healthy volunteers (n = 10) after supplementation with the tocotrienol-rich fraction (TRF)**. α-T3 was detected in all lipoprotein fractions starting from 2 h until 8 h. No α-T3 was detected at 0 h and 24 h (fasted states). Inset: AUC, area under the curve, 0-8 h (arbitrary units) is presented as mean ± SEM (n = 10) for α-T3 distribution in TRP, LDL and HDL.

In general, all T3 isomers (α-T3, γ-T3, δ-T3) except β-T3 were detected in all lipoprotein fractions (TRP, LDL, and HDL) following the TRF treatment although their concentration were significantly lower than α-T. Among the T3 isomers detected in TRP, LDL, and HDL, α-T3 was the major T3 isomer, followed by γ-T3 and δ-T3. In TRP, between 1.63 ± 0.57% to 4.65 ± 1.56% of total circulating plasma vitamin E was α-T3 (Figure 2). Starting at 2 h until 6 h, concentration of α-T3 was higher in TRP, compared to LDL and HDL. TRP α-T3 peaked at 5 h and declined thereafter. In LDL and HDL, although α-T3 peaked at 6 h, the TRP concentrations of this isomer remained higher at 6 h. Concentration of α-T3 in LDL and HDL was between 0.97 ± 0.32% to 2.96 ± 0.85% and 1.62 ± 0.48% to 3.13 ± 0.68% of total circulating plasma vitamin E, respectively.

**Figure 3:**
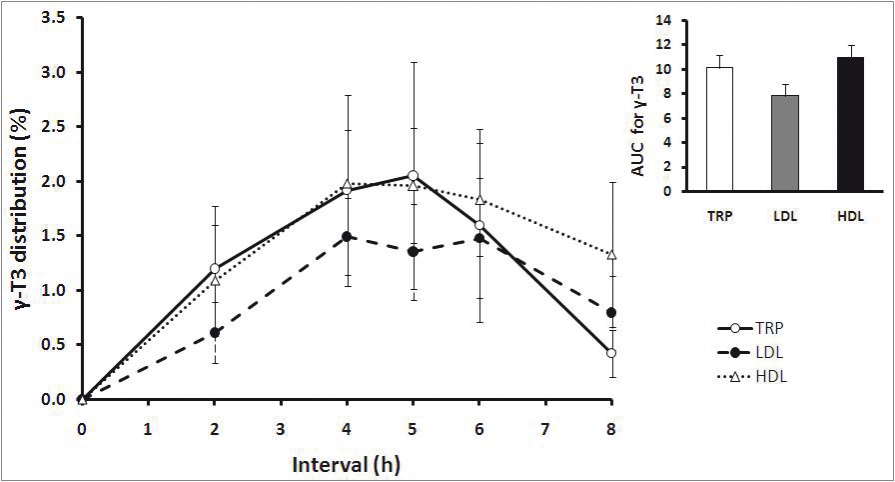
**Mean (± SEM) γ-tocotrienols (γ-T3) distributions in triacylglycerol rich particles, TRP (○); LDL (●); and HDL (Δ) of healthy volunteers (n = 10) after supplementation with the tocotrienol-rich fraction**. γ-T3 was detected in all lipoprotein fractions starting from 2 h until 8 h. No γ-T3 was detected at 0 h and 24 h (fasted states). Inset: AUC, area under the curve, 0-8 h (arbitrary units) is presented as mean ± SEM (n = 10) for γ-T3 distribution in TRP, LDL and HDL.

Starting from 2 h onwards, postprandial γ-T3 concentration in TRP and HDL increased gradually (Figure 3). γ-T3 concentration were similar in TRP and HDL, ranging between 0.42 ± 0.22% to 2.05 ± 1.04% and 1.09 ± 0.51% to 1.98 ± 0.49% of total circulating plasma vitamin E, respectively. γ-T3 in HDL peaked at 4 h whereas in TRP, γ-T3 peaked an hour later at 5 h, before steadily declining thereafter. Throughout the first 6-h postprandial period, concentration of γ-T3 was lower in LDL compared to TRP and HDL. Between 0.61 ± 0.28% to 1.49 ± 0.35% of total circulating plasma vitamin E was detected as γ-T3 in LDL. After 8 h, concentration of γ-T3 was highest in HDL, intermediate in LDL, and lowest in TRP.

**Figure 4:**
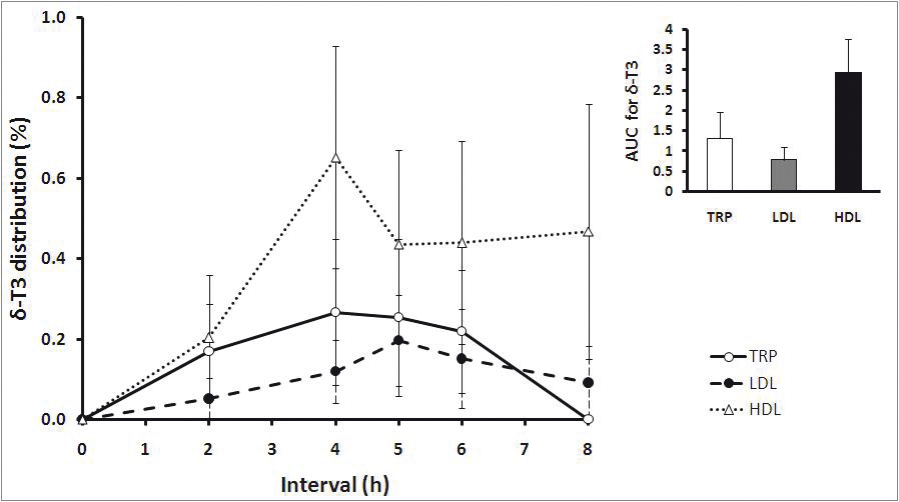
**Mean (± SEM) δ-tocotrienols (δ-T3) distributions in triacylglycerol rich particles, TRP (○); LDL (●) and HDL (Δ) of healthy volunteers (n = 10) after supplementation with the tocotrienol-rich fraction**. δ-T3 was detected in all lipoprotein fractions starting from 2 h until 8 h. No δ-T3 was detected at 0 h and 24 h (fasted states). Inset: AUC, area under the curve, 0-8 h (arbitrary units) is presented as mean ± SEM (n = 10) for δ-T3 distribution in TRP, LDL and HDL.

Following the 8-h postprandial period, among the lipoprotein fractions, HDL recorded the highest content of δ-T3 (Figure 4). Concentration of δ-T3 in the HDL was between 0.65 ± 0.28% and 0.47 ± 0.32% of total circulating plasma vitamin E. Starting from 2 h, HDL δ-T3 increased gradually and peaked at 4 h before plateauing. In LDL, δ-T3 increased steadily starting from 2 h, peaked at 5 h and declined thereafter. Up to 0.20 ± 0.11% of total circulating plasma vitamin E was detected as δ-T3 in LDL. Unlike LDL and HDL, δ-T3 in TRP was only detected starting from 2 h until 6 h postprandially. No δ-T3 was detected in TRP at 8 h. Concentration of δ-T3 in TRP was between 0.17 ± 0.12% to 0.27 ± 0.18% of total circulating plasma vitamin E.

## Discussion

Following both vitamin E treatments in the current study, α-T was the major vitamin E detected in circulating plasma and lipoproteins. All vitamin E isomers from dietary sources (including supplements) are absorbed and delivered to the liver, although only α-T is preferentially recognized by the α-tocopherol transfer protein (α-TTP) for incorporation into circulating plasma [25]. Other T (γ-T, δ-T) and T3 isomers (α-T3, γ-T3, δ-T3) are not preferentially utilized and are mostly excreted from circulation [26]. This is the main reason why α-T is the only vitamin E isomer that is currently used as the standard to estimate human vitamin E requirements [22]. However it is increasingly acknowledged that T3 and T serve different biological functions and bench marking only α-T to estimate human vitamin E requirements may no longer be the most accurate measure [3,5,8].

In the current study, supplementation with α-T or TRF resulted in significantly increased plasma α-T concentration compared to the baseline value. Furthermore, plasma total circulating α-T for the 24 h postprandial duration (described as AUC) was significantly higher after α-T treatment. This observation was however anticipated, due to the higher content of α-T administered (537 mg of α-T) from the α-T treatment compared to that of TRF (only 167 mg α-T).

In most human clinical and bioavailability studies of vitamin E, only the plasma and lipoprotein concentrations of α-T have been reported [27]. It would therefore be ideal if the concentrations of individual T and T3 are measured to gain new insight into the physiological roles of these vitamin E isomers in humans [27]. Evaluation of the metabolic response following T3 supplementation through plasma or serum concentration of T3 and α-T is advocated. Our present study shows that all T3 isomers (α-T3, γ-T3, δ-T3) were detected in plasma and lipoproteins following supplementation of TRF, although their concentration was significantly lower compared to that of α-T. These findings are in agreement with our previous postprandial observation [19] and several other human studies that examined the bio-kinetics [2,13,16,17,20] or physiological effects [9-12,14,15,21] of T3 supplementation.

Detection of individual vitamin E isomers in plasma, following postprandial challenge could assist in elucidating their preferential absorption into circulating blood. However, this may not be true for α-T, since the liver actively secretes α-T into circulating plasma and impacts final plasma concentration of this vitamin E isomer. α-T was detected in both fasted and postprandial states in the current study. T3 isomers, including α-T3, the major T3 isomer in TRF, however was not detected in the fasted state. Their occurrence throughout the postprandial state was apparent, only in significantly lower levels compared to α-T. Despite these observations, we note that T3 have been demonstrated to have biological functions well below plasma concentrations noted in this study (5, 8). Among the T3 isomers, the absorption rates appear in the order α-T3 > γ-T3 > δ-T3. These findings might explain the possibility of bio-discrimination between T and T3 isomers in humans. Such bio-discrimination has also been demonstrated in several animal studies. Ikeda et al. [28] demonstrated that α-T3 is preferentially absorbed into the lymphatic circulation compared to γ-T3 and δ-T3. Similar observations were found by Yap et al. [29] who investigated the influence of route of administration on the absorption and disposition of α-T3, γ-T3 and δ-T3 in rats. Of the 3 isomers, α-T3 achieved the highest concentration and AUC after an oral ingestion of T3. This was followed by γ-T3 and δ-T3. In humans, plasma concentrations of α-T3 were 2-fold higher than that of γ-T3, and almost 10 times higher than δ-T3 after supplementation with the same dose of T3 preparations [10]. Similar observations were also demonstrated in hypercholesterolemic subjects who received a high γ-T3 supplements that contained ≈4-fold concentration of γ-T3 than α-T3 [11].

Distribution of T3 isomers in lipoproteins also provides a better explanation of T3 absorption and transport in circulating plasma. In agreement with our previous observation [19], T3 were transported in TRP (chylomicrons + VLDL), LDL and HDL. Several mechanisms have been postulated to explain this observations [19] including the selectivity and affinity of hepatic α-TTP [30], the function of a specific protein carrier in transporting α-T3 in the intestinal cells [28], and differences in the methyl groups in the chromanol rings of T3 [29] that influenced the absorption rate of each T3 isomers [10]. Following its hepatic uptake, it would be intriguing to know whether nascent VLDL or HDL generated from the liver, is readily enriched in T3 from the liver itself. The role of HDL in transporting vitamin E has recently been identified as one of the primary mechanisms in vitamin E absorption in the fasted states [31].

The competitive uptake between isomers is only initiated following the hepatic uptake of vitamin E from chylomicrons, where the selectivity role of α-TTP is significant in transferring vitamin E into circulating VLDL [25]. The relative affinity of vitamin E isomers towards α-TTP has been demonstrated to be in the order of α-T (100%) > α-T3 (12%) > γ-T (9%) > δ-T (2%) [30]. This mechanism explains the occurrence of α-T as the major vitamin E isomer detected in TRP, LDL, and HDL, and the rapid disappearance of α-T3, γ-T3 and δ-T3 from circulating plasma and lipoproteins. Other physiological factors such as bile, urinary and fecal excretion that may influence the rapid disappearance of T3 has also been postulated [19,32]. The exchange of T3 between circulating chylomicron, VLDL, LDL, and HDL has also been suggested to explain their distribution in the lipoproteins [19,20].

There is no bio-discrimination between T and T3 during intestinal absorption after dietary intake of vitamin E [26,33-35]. However, the rapid disappearance of T3 may be associated with its preferential utilization in humans (8, 25, 34). In the current and previous [19,20] studies, the amount of T3 absorbed into TRP was very low. This observation may indicate the possibility of bio-discrimination of T3, prior to the intestinal absorption. Although mechanism for the preferential absorption of T3 is difficult to describe, it has been suggested that the complexity of T3 absorption is probably due to the difference in their micellar solubility, affinity for intestinal brush border membranes, transport in enterocytes, incorporation into chylomicrons, or a combination of these processes [28]. Besides, there might be variability in the mucosal handling of vitamin E that could affect their intestinal absorption [36]. Although we did not separate chylomicrons and VLDL from TRP fraction to differentiate the T3 uptake from intestine by chylomicrons and from liver by VLDL, recent findings from Abuasal et al. [37] demonstrated that there was an inverse relationship between intestinal uptake of γ-T3 and their concentration in the intestinal lumen. Therefore, any elevation of γ-T3 concentration in the lumen would likely reduce the amount of γ-T3 transported into the enterocytes. However, no investigations on other T3 isomer were carried out. The intestinal absorption of T3, as well as T still merits further investigations, since their mechanism has not been fully described [25]. In rats, dietary vitamin E including T3 are converted to their metabolite by CYP-dependent pathway in the intestine during absorption. This could likely regulate T3 concentration in plasma and tissue [38]. Yet, excess intake of T3 has been observed to lead excretion of α-T3 and γ-T3 into bile, before both T3 isomers were metabolized into α- and γ-CEHC derivatives [32].

The postprandial dose response effect of vitamin E in humans has basically been evaluated from the plasma and lipoproteins profiles of α-T and γ-T [39-41]. Surprisingly, no such evidence exists for T3, although T3 always positively imaged as a superior antioxidant compared to T [2,8]. In the previous study [19], we investigated the postprandial response after 1011 mg TRF supplementation. In fact, this dose used was higher than the Tolerable Upper Limit Intake (UL) for vitamin E [22]. One of the rationale of conducting the current study was to investigate whether supplementation with 526 mg TRF would resulted a similar postprandial response, in comparison to the dose used in the previous study [19], since concentrations of vitamin E in plasma can only be raised maximally two to three-fold after supplementation [39]. Plasma α-T3, γ-T3 and δ-T3 response after TRF treatment in the current study were not significantly different from the previous study. Additionally, α-T and γ-T concentrations in plasma, TRP, LDL and HDL were not apparent between both TRF treatments. However, observations in lipoprotein fractions still remains to be elucidated. In HDL, starting from 4 h to 6 h postprandial, α-T3 concentration after 526 mg TRF treatment were significantly lower compared to the 1011 mg TRF treatment. These observations merits further investigation since the transportation of vitamin E by HDL may possibly be influenced by supplementation dose and was not affected by amount of dietary fat intake [31]. In both postprandial studies, the amount of dietary fat in the test breakfast consumed before TRF supplementation was standardized.

Several studies have suggested the effectiveness of T3 as a hypocholesterolemic agent in lowering plasma or serum total cholesterol in humans [15,42]. Nevertheless, it is questionable why the effectiveness of T3 in lowering plasma total cholesterol has not been compared with α-T, since α-T has been recognized as the only form utilized to estimate human vitamin E requirements. Furthermore, the effectiveness of T3 in humans was only compared with a placebo treatment in most studies [10,15,18,21,42,43]. Although in several studies, physiological effects of T3 was compared with α-T, the concentration of α-T in the control preparations or supplements was very low [9,11,44] Unlike our previous observation [19] where supplementation with 1011 mg palm TRF or 1074 mg α-T resulted in significant lowering of plasma postprandial total cholesterol, supplementation with 526 mg palm TRF or 537 mg α-T in the current study did not demonstrated any hypocholesterolemic effect. Several postulations have been discussed to explain the inability of T3 to lower plasma or serum cholesterol in humans such as the higher content of T in the T3 supplements, *in vivo *bio-conversion of T3 to α-T, and very low concentration of T3 that did not reach the pharmacologically effective level in plasma [45].

## Conclusions

In conclusions, T3 isomers (α-T3, γ-T3, and δ-T3) were present in the circulating plasma and lipoproteins (TRP, LDL, and HDL) after T3 supplementation. As postulated, T3 concentrations were significantly lower than α-T. Low absorption into the circulation could affect the physiological effects of T3, as indicated by their inability to lower plasma cholesterol in the current postprandial study. However, it is somewhat reassuring that even at the low concentration of circulating T3 in plasma (approximately 4 nM) T3 could still have beneficial biological functions including that of neuroprotection as demonstrated by other workers [46].

## Abbreviations

AUC: area under the curve; CEHC: carboxy-ethyl-hydroxy-chroman; CYP: cytochrome P450; d: day; h: hour; MANOVA: multiple analysis of variance; T: tocopherols; T3: tocotrienols; TRF: tocotrienol-rich fraction; UL: upper tolerable intake; α-T: alpha-tocopherol; α-T3: alpha-tocotrienol; β-T3: beta-tocotrienol; δ-T3: delta-tocotrienol; γ-T: gamma-tocopherol; γ-T3: gamma-tocotrienol.

## Competing interests

The authors declare that they have no competing interests.

## Authors' contributions

The contribution of each author was as follows: SF undertook the overall management of the study and most of the laboratory and statistical analysis and drafting of the manuscript. RMN developed the methods for vitamin E HPLC analysis and other related analytic procedures. HMC contributed to the design and subsequent finalization of the manuscript. KS was the overall researcher in charge of the study, having designed the study protocols, primed the laboratory and statistical techniques, and contributed intellectually to the final manuscript. All authors read and approved the final manuscript.

## References

[B1] Serbinova EA Kagan V Han D Packer L Free radical recycling and intramembrane mobility in the antioxidant properties of α-tocopherol and α-tocotrienol Free Radic Biol Med 1991 10 263 275 10.1016/0891-5849(91)90033-Y 1649783

[B2] Suarna C Hood RL Dean RT Stocker R Comparative antioxidant activity of tocotrienols and other natural lipid soluble antioxidants in homogenous system, and in rat and human lipoproteins Biochim Biophys Acta 1993 1166 163 170 8443232 10.1016/0005-2760(93)90092-n

[B3] Theriault A Jun-Tzu C Wang Q Gapor A Adeli K Tocotrienol: a review of its therapeutic potential Clin Biochem 1999 32 309 319 10.1016/S0009-9120(99)00027-2 10480444

[B4] Packer L Weber SU Rimbach G Molecular aspects of α-tocotrienol antioxidant action and cell signaling J Nutr 2001 131 Suppl 369 373 10.1093/jn/131.2.369S 11160563

[B5] Sylvester PW Theriault A Role of tocotrienols in the preventive of cardiovascular disease and breast cancer Curr Top Nutraceutical Res 2003 1 121 136

[B6] Kerckhofts DAJM Brouns F Honstra G Mensink RP Effects on the human serum lipoprotein profile of β-glucan, soy protein and isoflavones, plant sterols and stanols, garlic and tocotrienols J Nutr 2002 132 2494 2505 12221200 10.1093/jn/132.9.2494

[B7] Schaffer S Müller WE Eckert GP Tocotrienols: constitutional effects in aging and disease J Nutr 2005 135 151 154 15671205 10.1093/jn/135.2.151

[B8] Sen CK Khanna S Roy S Tocotrienols: vitamin E beyond tocopherols Life Sciences 2006 78 2088 2098 10.1016/j.lfs.2005.12.001 16458936 PMC1790869

[B9] Mensink RP van Houwelingen AC Kromhout D Honstra G A vitamin E concentrate rich in tocotrienols had no effect on serum lipids, lipoproteins, or platelet function in men with mildly elevated serum lipid concentrations Am J Clin Nutr 1999 69 213 219 9989682 10.1093/ajcn/69.2.213

[B10] O'Byrne D Grundy S Packer L Devaraj S Baldenius K Hoppe PP Kramer K Jialal I Traber MG Studies of LDL oxidation following α-, γ-, or δ-tocotrienyl acetate supplementation of hypercholesterolemic humans Free Radic Biol Med 2000 29 834 845 10.1016/S0891-5849(00)00371-3 11063909

[B11] Mustad VA Smith CA Ruey PP Edens NK DeMichele SJ Supplementation with 3 compositionally different tocotrienol supplements does not improve cardiovascular disease risk factors in men and women with hypercholesterolemia Am J Clin Nutr 2002 76 1237 1243 12450888 10.1093/ajcn/76.6.1237

[B12] Radhakrishnan AK Lee AL Wong PK Kaur J Aung H Nesaretnam K Daily supplementation of tocotrienol-rich fraction or α-tocopherol did not induce immunomodulatory changes in healthy human volunteers Br J Nutr 2009 101 810 815 10.1017/S0007114508039998 18702848

[B13] Hayes KC Pronczuk A Liang JS Difference in the plasma transport and tissue concentration of tocopherols and tocotrienols: observations in humans and hamsters Proc Soc Exp Biol Med 1993 202 353 359 8437992 10.3181/00379727-202-43546

[B14] Wahlqvist ML Krivokuca-Bogetic Z Sam Lo C Differential serum responses of tocopherols and tocotrienols during vitamin supplementation in hypercholesterolaemic individuals without change in coronary risk factors Nutr Res 1992 12 Suppl 181 201

[B15] Qureshi AA Bradlow BA Salser WA Brace LD Novel tocotrienols of rice bran modulate cardiovascular disease risk parameters of hypercholesterolemic humans J Nutr Biochem 1997 8 290 298 10.1016/S0955-2863(97)89667-2

[B16] Yap SP Yuen KH Wong JW Pharmacokinetics and bioavailability of α-, γ- and δ-tocotrienols under different food status J Pharm Pharmacol 2001 53 67 71 11206194 10.1211/0022357011775208

[B17] Yap SP Yuen KH Influence of lipolysis and droplet size on tocotrienol absorption from self-emulsifying formulations Int J Pharm 2004 281 67 78 10.1016/j.ijpharm.2004.05.015 15288344

[B18] Rasool AHG Yuen KH Yusoff K Wong AR Rahman ARA Dose dependent elevation of plasma tocotrienol level and its effect on arterial compliance, plasma total antioxidant status, and lipid profile in healthy humans supplemented with tocotrienol rich vitamin E J Nutr Sci Vitaminol 2006 52 473 478 10.3177/jnsv.52.473 17330512

[B19] Fairus S Nor RM Cheng HM Sundram K Postprandial metabolic fate of tocotrienol-rich vitamin E differs significantly from that of α-tocopherol Am J Clin Nutr 2006 84 835 842 17023711 10.1093/ajcn/84.4.835

[B20] Khosla P Patel V Whinter JM Khanna S Rakhkovskaya M Roy S Sen CK Postprandial levels of the natural vitamin E tocotrienol in human circulation Antioxid Redox Signal 2006 8 1059 1068 10.1089/ars.2006.8.1059 16771695

[B21] Rasool AHG Rahman ARA Yuen KH Wong AR Arterial compliance and vitamin E blood levels with a self emulsifying preparation of tocotrienol rich vitamin E Arch Pharm Res 2008 31;1212 1217 10.1007/s12272-001-1291-5 18806966

[B22] Food and Nutrition Board, Institute of Medicine Dietary reference intakes for vitamin C, vitamin E, selenium and carotenoids 2000 Washington DC: National Academy Press 25077263

[B23] Sundram K Hayes KC Siru OH Dietary palmitic acid results in lower serum cholesterol than does a lauric-myristic acid combination in normolipemic humans Am J Clin Nutr 1994 59 841 846 8147328 10.1093/ajcn/59.4.841

[B24] Sundram K Nor RM Armstrong D From analysis of tocotrienols in different sample matrixes by HPLC Methods in Molecular Biology, Oxidative Stress Biomarkers and Antioxidant Protocols 2002 186 Totowa, New Jersey: Humana Press Inc 221 232 10.1385/1-59259-173-6:221 12013770

[B25] Traber MG Vitamin E regulatory mechanisms Annu Rev Nutr 2007 27 347 362 10.1146/annurev.nutr.27.061406.093819 17439363

[B26] Traber MG Kayden HJ Preferential incorporation of alpha-tocopherol vs gamma-tocopherol in human lipoproteins Am J Clin Nutr 1989 49 517 526 2923084 10.1093/ajcn/49.3.517

[B27] Schwenke DC Vitamin E to prevent cardiovascular disease: pill or dietary package? Curr Opin Lipidol 2007 18 467 469 10.1097/MOL.0b013e32825fea3a 17620864

[B28] Ikeda I Sasaki E Sugano M Lymphatic transport of α-, γ- and δ-tocotrienols and α-tocopherol in rats Int J Vit Nutr Res 1996 66 217 221 8899454

[B29] Yap SP Yuen KH Lim AB Influence of route of administration on the absorption and disposition of α-, γ- and δ-tocotrienols in rats J Pharm Pharmacol 2003 55 53 58 12625867 10.1111/j.2042-7158.2003.tb02433.x

[B30] Hosomi A Arita M Sato Y Kiyose C Ueda T Igarashi O Arai H Inoue K Affinity for α-tocopherol transfer protein as a determinant of the biological activities of vitamin E analogues FEBS Letters 1997 409 105 108 10.1016/S0014-5793(97)00499-7 9199513

[B31] Anwar K Iqbal J Hussain MM Mechanisms involved in vitamin E transport by primary enterocytes and *in vivo *absorption J Lipid Res 2007 48 2028 2038 10.1194/jlr.M700207-JLR200 17582142

[B32] Lodge JK Ridllington J Leonard S Vaule H Traber MG α- and γ-tocotrienols are metabolized to carboxyethyl-hydroxychroman derivatives and excreted in human urine Lipids 2001 36 43 48 10.1007/s11745-001-0666-z 11214728

[B33] Traber MG Burton GW Hughes L Ingold KU Hidaka H Malloy M Kane J Hyams J Kayden HJ Discrimination between forms of vitamin E by humans with and without genetic abnormalities of lipoprotein metabolism J Lipid Res 1992 33 1171 1182 1431596

[B34] Kayden HJ Traber MG Absorption, lipoprotein transport, and regulation of plasma concentrations of vitamin E in humans J Lipid Res 1993 34 343 358 8468520

[B35] Kiyose C Muramatsu R Kameyama Y Ueda T Igarashi O Biodiscrimination of α-tocopherol stereoisomers in humans after oral administration Am J Clin Nutr 1997 65 785 789 9062530 10.1093/ajcn/65.3.785

[B36] Jeanes YM Hall WL Ellard S Lee E Lodge JK The absorption of vitamin E is influenced by the amount of fat in a meal and the food matrix Br J Nutr 2004 92 575 579 10.1079/BJN20041249 15522126

[B37] Abuasal A Sylvester PW Kaddoumi A Intestinal absorption of γ-tocotrienol is mediated by Niemann-Pick C1-Like 1: *in situ *rat intestinal perfusion studies Drug Metab Dipos 2010 38 939 945 10.1124/dmd.109.031567 20207946

[B38] Abe C Uchida T Ohta M Ichikawa T Yamashita K Ikeda S Cytochrome P450-dependent metabolism of vitamin E isoforms is a critical determinant of their tissue concentration in rats Lipids 2007 42 637 645 10.1007/s11745-007-3064-2 17520307

[B39] Traber MG Rader D Acuff RV Ramakrishnan R Brewer HB Kayden HJ Vitamin E dose-response studies in humans with use of deuterated RRR-α-tocopherol Am J Clin Nutr 1998 68 847 853 9771861 10.1093/ajcn/68.4.847

[B40] Meydani M Cohn JS Macauley JB McNamara JR Blumberg JB Schaefer EJ Postprandial changes in the plasma concentration of α- and γ-tocopherol in human subjects fed a fat-rich meal supplemented with fat-soluble vitamins J Nutr 1989 119 1252 1258 2795239 10.1093/jn/119.9.1252

[B41] Borel P Mekki N Boirie Y Partier A Grolier P Alexandre-Gouabau MC Beaufrere B Armand M Lairon D Azais-Braesco V Postprandial chylomicron and plasma vitamin E responses in healthy older subjects compared with younger ones Eur J Clin Invest 1997 27 812 821 10.1046/j.1365-2362.1997.1960744.x 9373758

[B42] Baliarsingh S Beg ZH Ahmad J The therapeutic impacts of tocotrienols in type 2 diabetic patients with hyperlipidemia Atherosclerosis 2005 182 367 374 10.1016/j.atherosclerosis.2005.02.020 16159610

[B43] Qureshi AA Sami SA Salser WA Dose-dependent suppression of serum cholesterol by tocotrienol-rich fraction (TRF_25_) of rice bran in hypercholesterolemic humans Atherosclerosis 2002 161 199 207 10.1016/S0021-9150(01)00619-0 11882333

[B44] Qureshi AA Bradlow BA Brace L Manganello J Peterson DM Pearce BC Wright JJK Gapor A Elson CE Response of hypercholesterolemic subjects to administration of tocotrienols Lipids 1995 30 1171 1177 10.1007/BF02536620 8614309

[B45] Yu SG Thomas AM Gapor A Tan B Qureshi N Qureshi AA Dose-response impact of various tocotrienols on serum lipid parameters in 5-week-old female chickens Lipids 2006 41 453 461 10.1007/s11745-006-5119-1 16933790

[B46] Sen CK Rink C Khanna S Palm oil derived natural vitamin E α-tocotrienol in brain disease and health J Am Coll Nutr 2010 29 314S 323S 20823491 10.1080/07315724.2010.10719846 PMC3065441

